# Prokineticin Receptor 1 as a Novel Suppressor of Preadipocyte Proliferation and Differentiation to Control Obesity

**DOI:** 10.1371/journal.pone.0081175

**Published:** 2013-12-04

**Authors:** Cécilia Szatkowski, Judith Vallet, Mojdeh Dormishian, Nadia Messaddeq, Phillippe Valet, Mounia Boulberdaa, Daniel Metzger, Pierre Chambon, Canan G. Nebigil

**Affiliations:** 1 Institute of Research and Biotechnology of Strasbourg, Centre national de la recherche scientifique, UMR7242, University of Strasbourg, Medalis/Labex, Drug Discovery Center, Illkirch, France; 2 Institute of Genetic and Molecular and Cellular Biology, Centre national de la recherche scientifique UMR7104, Institut National de la Santé et de la Recherche Médicale U964, University of Strasbourg, Illkirch, France; 3 Institutes of Cardiovascular and Metabolic Diseases, Institut National de la Santé et de la Recherche Médicale–University of Paul Sabatier UMR 1048, Toulouse, France; University of Minnesota - Twin Cities, United States of America

## Abstract

**Background:**

Adipocyte renewal from preadipocytes occurs throughout the lifetime and contributes to obesity. To date, little is known about the mechanisms that control preadipocyte proliferation and differentiation. Prokineticin-2 is an angiogenic and anorexigenic hormone that activate two G protein-coupled receptors (GPCRs): PKR1 and PKR2. Prokineticin-2 regulates food intake and energy metabolism via central mechanisms (PKR2). The peripheral effect of prokineticin-2 on adipocytes/preadipocytes has not been studied yet.

**Methodology/Principal Findings:**

Since adipocytes and preadipocytes express mainly prokineticin receptor-1 (PKR1), here, we explored the role of PKR1 in adipose tissue expansion, generating PKR1-null (PKR1^−/−^) and adipocyte-specific (PKR1^ad−/−)^ mutant mice, and using murine and human preadipocyte cell lines. Both PKR1^−/−^ and PKR1^ad−/−^ had excessive abdominal adipose tissue, but only PKR1^−/−^ mice showed severe obesity and diabetes-like syndrome. PKR1^ad−/−)^ mice had increased proliferating preadipocytes and newly formed adipocyte levels, leading to expansion of adipose tissue. Using PKR1-knockdown in 3T3-L1 preadipocytes, we show that PKR1 directly inhibits preadipocyte proliferation and differentiation. These PKR1 cell autonomous actions appear targeted at preadipocyte cell cycle regulatory pathways, through reducing cyclin D, E, cdk2, c-Myc levels.

**Conclusions/Significance:**

These results suggest PKR1 to be a crucial player in the preadipocyte proliferation and differentiation. Our data should facilitate studies of both the pathogenesis and therapy of obesity in humans.

## Introduction

Obesity causes many serious diseases such as type-2 diabetes mellitus, cardiovascular diseases and certain types of cancer, and has contributed to increases in mortality and morbidity rates [1]. Identification of factors regulating white fat tissue growth provides an important strategy to combat these diseases. Obesity is characterized by an expansion of adipose tissue mass due to hypertrophy, an increase in adipocyte size [2], and hyperplasia, an increase in cell number [3]. The expended adipose tissue plays a key role for the induction of insulin resistance commonly seen in obesity [4]. The adipocytes increase in size is due to lipid accumulation [5]. Mature adipocytes are postmitotic [6]. Thus, adipocyte hyperplasia in adults requires the generation of new adipocytes from precursor cells (preadipocytes) and stem cells resident in the stromal-vascular compartment of white adipose tissues (WATs). Preadipocytes are capable of proliferating and differentiating into an adipose deposit [7]. Stimulation of the proliferation of these cells may therefore result principally in an increase in adipocyte number. In mice, preadipocytes can proliferate and subsequently differentiate into mature adipocytes [8]. Thus, understanding the mechanisms controlling preadipocyte proliferation and conversion to adipocyte provides insights into the etiology and prevention of obesity and its associated pathologies.

Prokineticin-2 is a potent angiogenic [9] and anorexigenic hormone [10]. It binds two similar G protein-coupled receptors (GPCRs): PKR1 and PKR2 [11]. This hormone, which is widely distributed in mammalian tissues [12], has HIF-1 binding sites and is induced by low oxygen levels [13]. It is involved in diverse effects in peripheral systems, including angiogenesis in the ovary, testis [14], [15] and heart [16]. It also stimulates hematopoiesis [17] and neurogenesis [18]. Prokineticins induce the differentiation of murine and human bone marrow cells into the monocyte/macrophage lineage [19] and activate monocyte proliferation and differentiation [20] and macrophage migration [21]. Prokineticin-2/PKR1 restores the pluripotency of epicardial progenitor cells and triggers the differentiation of endothelial and vascular smooth muscle cells [16]. By binding to PKR1, prokineticin-2 directly promotes angiogenesis, by activating MAPK and Akt [16].

Prokineticin-2 [22] is involved in appetite suppression [10] and energy homeostasis, thermoregulation (15), via a direct hypothalamic mechanism [23]. The intracranial or peripheral injection of prokineticin-2 has been shown to reduce food intake and body weight in lean and obese mice at the levels of hypothalamus (central effect via PKR2) [10] and dorsal vagal complex (peripheral effects via PKR1) [24]. However, the role of prokineticin-2 and its receptors in adipocyte and preadipocyte function is unknown.

PKR1 is the principal receptor expressed by preadipocytes and adipocytes [12]. In this study, we explored the role of PKR1 in adipocyte function, in vivo using PKR1-null (PKR1^−/−^) and adipocyte-specific (PKR1^ad−/−)^ mutant mice, and in vitro, using murine (3T3-L1) and human preadipocyte cell lines (SGBS). Here we described multiple PKR1 functions regulates preadipocyte proliferation and differentiation, controlling adipose tissue expansion.

## Materials and Methods

### Ethics statement

The animal study was approved by the Animal Care and Use, and ethics committees of the Perfecture du Bas-Rhin (Permit Number: B67–274) with the recommendations in the Guide for the Care and Use of Laboratory Animals of the French Animal Care Committee, with European regulation-approved protocols. The animal experimentation and housing were conducted at the accredited Animal Experimentation and housing Facility of the Institut de recherche de l′Ecole de biotechnologie de Strasbourg (Register number: C67-218-19).

The human study was approved by the ethics committee of Toulouse-Rangueil and Nancy-J. d'Arc Hospitals. Human adipose tissues were collected according to the guidelines of the Ethical Committee of Toulouse-Rangueil and Nancy-J. d'Arc Hospitals. All subjects gave their informed consent to participate to the study and investigations were performed in accordance with the declaration of Helsinki as revised in 2000 (http://www.wma.net/e/policy/b3.htm). Human subcutaneous adipose tissue samples were obtained from patients undergoing abdominal lipectomy for plastic surgery or before a bariatric surgery and immediately frozen in liquid nitrogen and stored at −80°C. Written informed consent was obtained from all subjects and samples were coded and treated anonymously.

### Genetically manipulated animal models

Male PKR1^−/−^ mice in C57BL/6 gene background were originally made by homologous recombination [25]. For diet-induced obesity, 6-week-old PKR1-deficient mice and their wild-type littermates (C57BL/6) were kept in micro isolation cages and fed a high-fat diet containing 60% fat or normal cow diet (5% fat) (Research Diet, New Brunswick, NY) for 34 weeks. PKR1 floxed mice [25] and *aP2-Cre*ERT2 [26] on a C57BL6/J background were intercrossed to generate the following genotypes: PKR1L2/L2 × *aP2-Cre*ERT2(Tg/0) and PKR1L2/L2 × *aP2-Cre*ERT2(0/0). Mice in which PKR1 was inactivated after tamoxifen injection will be referred to as PKR1^ad−/−^ and their controls will be referred to as L2/L2. Tamoxifen was injected 1 mg/day (i.p.) for 5 days to the L2/L2 and PKR1^ad−/−^ mice at the 3 weeks of age. The second tamoxifen injection was realized at the age of 13 weeks. All the analyses were performed when the mice were 24 and 40 weeks old. Mice were placed in metabolic cages (1 mouse/cage) and acclimated for 2 days before the experiment. The body weight, water and food intake, and urine volume were performed daily for an additional 3 days. The primers utilized in these studies for the genotyping analyses were shown in the Table 1.

**Table 1:** Oligonucleotides used for PCR analyses.

Genes	Sequence Forward (5′ ->3′)	Sequence Reverse (5′ ->3′)
Beta actin	CATCTTGGCCTCACTGTCCA	GGGCCGGACTCATCGTACT
PPAR alpha	ACGATGCTGTCCTCCTTGATG	GTGTGATAAAGCCATTGCCGT
PPAR gamma	CGAGAAGGAGAAGCTGTTGG	GAAACTGGCACCCTTGAAAA
C/EBP alpha	CAAGAACAGCAACGAGTACCG	GTCACTGGTCAACTCCAGCAC
Adiponectin	AATCCTGCCCAGTCATGCCGAAG	TCTCCAGGAGTGCCATCTCTGCCATC
Resistin	AGAAGGCACAGCAGTCTT	TGTCCAGTCTATCCTTGC
Cyclin D1	GAGATTGTGCCATCCATGC	CTCCTCTTCGCACTTCTGCT
Cyclin E1	TTCTGCAGCGTCATCCTCT	TGGAGCTTATAGACTTCGCACA
Cdk-2	CACAGCCGTGGATATCTGG	CATGGTGCTGGGTACACACT
c-Myc	CCTAGTGCTGCATGAGGAGA	TCTTCCTCATCTTCTTGCTCTTC
HIF1 alpha	TACAAGGCAGCAGAAACCTAC	TGTGCAATTGTGGCTACC
mPKR1	GCCATTGCCATTGACAGGTA	TGGTGAAGTAGGCAGCTGGA
UCP	CTTGTCAACACTTTGGAAAGG	CCTTGGTGTACATGGACATC
GLUT 4	TGTTTTGAAGAACGGATAGG	CGGATTTCTTGAGTTCAAGG
TNFalpha	ACGGCATGGATCTCAAAGAC	AGATAGCAAATCGGCTGACG
hPK2	CTTGCCTCTTCCACCTCAAA	TGCAAGAGGAGGGAAGAGAA
hPK-R1	CGGCATTGGAAACTTCATCT	GATGAGCAGGTTGGTGAGGT
Cre	ATC TTC CAG CAG GCG CAC CAT TGC CCC TGT	TGA CGG TGG GAG AAT GTT AAT CCA TAT TGG
L2/L2	GAC TGG ACA TCT AGT GGT AGT CAG G	GGG TGT GAG GTG GGA TTA AGT CAC

### Glucose tolerance test (GTT) and insulin tolerance test (ITT)

Prior to studies, mice were fasted for 5 h. For GTT, mice received an intraperitoneal injection of glucose (1 mg/g body weight). In ITT studies, mice received an intraperitoneal injection of 0.75U of insulin per kg of body weight according to methods described elsewhere [27]. Blood samples were collected from the tail vein (tail-snip technique) at various times after the glucose or insulin load, as indicated. Blood glucose was immediately determined on a Contour blood glucose monitoring system (Bayer).

### Insulin stimulation of Akt activity detected by Western Blot assay

Animals were anaesthetized with 1% pentobarbital and were followed by an IP injection of insulin (150 mU/g, Umaline®, Rapide). 0 and 20 min after injection mice were scarified and the adipose tissues were harvested for protein extraction. Extracted proteins were transferred to nitrocellulose membranes and immunoblotted with the Phospho Akt (Ser-473 and Thr 308) Antibody kit (Cell Signaling Technology), following incubation with horseradish peroxidase-conjugated goat anti-rabbit IgG. Phosphorylated protein was visualized by enzyme-linked chemiluminescence (Amersham Biosciences) and quantified by scanning laser densitometry, normalizing to total amounts of the Akt proteins.

### Histological and electron microscopy analyses

Organs were removed from 24 or 40 week-old mice, dissected and frozen for the cutting of frozen sections (5 µm), which were stained with Mallory tetrachrome. For electron microscopy, all organs were fixed by and embedded in epoxy resin according to methods described elsewhere [27].

Adipocyte diameters were analyzed on cryosectioned adipose tissue (3 independent sections) and 6–7 pictures were taken for each section. We counted more than 50 adipocytes per piece of WAT, resulting in a total of 3–400 adipocytes counted per mouse (total 3 mice). Quantification of adipocyte size was done with ImageJ software (http://rsbweb.nih.gov/ij/). All images were converted into binary files using a unique threshold value that separated positively labeled cells from background.

For succinic dehydrogenase (SDH) stain, excised adipose tissues were snap frozen in OCT compound (Sakura) and sectioned at 50 µm. Subsequent staining procedure was performed by standard protocols [28]. The staining intensity was quantified using ImageJ program. Microscope images were converted to gray scale and inverted. Pixels between the threshold 95∼255 were selected to avoid the background signal, and the integrated densities were measured.

### Immunostaining analyses

Frozen tissue sections for the immunofluorescence staining of structural proteins were fixed, blocked and stained with primary antibodies against PECAM-1, (Santa Cruz), PKR1 (IGBMC, Illkirch), Collagen type VI (abcam pAb to collagen VI), CD 68 (Serotec), pref-1 (Abcam) and PPARγ (Santa Cruz). Antibody binding was detected by incubation with Fluorescein, Alexa 555-, Alexa 488- and Alexa 594-conjugated secondary antibodies [27]. Finally, the nuclei were stained with DAPI. Fluorescence was analyzed on a Leica fluorescence microscopy. The quantification was done with Image J software, by counting 20 fields at ×40 magnification per tissue section for each group of mice (at least 3 mice). The measurements of immunofluorescence intensity were performed with a Leica TCSNT confocal microscope or fluorescent microscope. Signal intensity was quantified on digitalized images and calculated as the product of averaged pixel intensity per high-power field or per dapi positive total nucleus number. Some of the fluorescent signals were obtained with confocal microscope and images were controlled by LEICA software. The laser was chosen according to the sample and compared pixel intensity and pixel distribution. These data were analyzed using the pixel data from which background intensity was subtracted.

### Evaluation of proliferation and apoptosis

Cryosectioned mice adipose tissue samples or cultured cells were obtained and then, apoptosis was detected by the TdT-mediated dUTP nick end-labeling (TUNEL) assay utilizing Apoptag flurescein in situ apoptosis detection kit (Milipore) according to the manufacturer's protocol [16]. Proliferation was evaluated with Ki67 Labeling (Santa Cruz), as previously described protocol [27]. Cryosectioned mice adipose tissue samples or cells were fixed, blocked and stained with primary antibodies against Ki67. Antibody binding was detected by incubation with Fluorescein, Alexa 488-conjugated secondary antibody. Cells were scored for Tunel or Ki67-positive nuclei corresponding to DAPI stained nucleus in the each high power field (20 minimum) containing at least 30 nuclei at ×40 magnification.

For FACS analyses, epididymal WATs of 40-week-old male mice were digested with 1 mg/ml collagenase Type IV (Sigma-Aldrich), filtered through sterilized lens paper, and centrifuged. Enriched adipocytes floating on the top were removed. Cell pellets were permeabilized (20 h at −20°C in 70% ethanol). At least 1×10^5^ cells in PBS/0.5% BSA/2 mmol/liter EDTA were incubated with fluorescein isothiocyanate (FITC)-conjugated Ki-67 antibody. The labeled cells were analyzed by multiparameter flow cytometry using a FACSCalibur flow cytometer and the CellQuest Pro software (BD Bioscience).

### Cell counting

Murine preadipocytes, 3T3-L1 cells (10^5^) were obtained from American Type Culture Collection, and cultured in DMEM, for 48 h then cell number was counted. In the other settings growth capacity of 3T3-L1 cells was determined in which 6×10^4^ cells were plated in DMEM containing 0.5 or 10% FBS in the presence or absence of prokineticin-2 (peprotech) (1, 5, 10 nM). Total cell number per well was daily counted from d 0–4 with a hemocytometer [29]. Note that maximum effect was observed with prokineticin-2 at 5 nM concentration.

### Adipocyte differentiation by oil red O staining

Confluent 3T3-L1 were differentiated into adipocytes by the DMEM supplemented with 0.5 mM isobutylmethylxanthine (IBMX) (Sigma-Aldrich), 1 µM dexamethasone (Sigma-Aldrich), and 10 µg/ml insulin (Sigma-Aldrich), as previously described [30]. After 2 days the medium was replaced with the medium containing only 10 µg/ml insulin. The human cell strain, derived from an adipose depot of an infant with SimpsoneGolabieBehmel syndrome (SGBS), was cultured as follows: briefly, confluent cells (day 0) were induced to differentiate in DMEM/Ham's F12 (1∶1) medium containing 0.01 mg/ml transferrin, 100 nM cortisol, 0.2 nM triiodothyronine, and 20 nM insulin. To trigger the differentiation, 25 nM dexamethasone, 500 mM IBMX and 2 mM rosiglitazone were present from day 0 to day 4. Intracellular accumulation of lipid droplets became clearly evident at day 10. The cells were stained with Oil Red O (0.5 g in 100 ml isopropanol) at the day 8 for 3T3-L1, at the day 10 for SGBS cells, to visualize the lipid accumulation. In the other settings 3T3-L1 cells were treated with prokineticin -2 (5 nM) 10 h before adipogenic stimuli, because the entry into S phase by 10% serum occurs within 10 h. For quantitative analysis of Oil Red O staining, oil red positive cells were detected and calculated as % of total dapi positive cells in the at least 20 random fields from each samples, using an inverted phase contrast microscope (Leica). Images were captured at a magnification of 20× or 40× with a digital microscope camera system. ImageJ software (National Institutes of Health) was used to convert bright field (24 bit) images of Oil red O stainings to 8 bit images. Threshold values were chosen that maximize selection of the Oil red O positive tissue while minimizing background interference. Thus, the total number of lipid drops from each image (percent) was quantified and normalized by dapi positive cell number [31]. Differentiation of adipose cells was also assessed by measuring triacylglycerol content of the homogenates, using the Triglyceride Enzymatic PAP150 kit (BioMe'rieux, Marcy l′Etoile, France) [31].

### RNA interference

The RNA interference technique was used for down-regulating PKR1 gene expression as previously described [32]. Transfection was performed by using siPORT Amine transfection reagent (Ambion, Austin, TX, USA) with 10 nM siRNA for mouse PKR1 (Ambion siRNA #181827), according to the manufacturer's instructions (Ambion). Negative control siRNA transfection was also performed by using non-specific siRNA (Ambion). After 48 hours medium was replaced with induction medium to induce differentiation into mature adipocyte or to isolate RNA for detection of PKR1 levels by PCR. In this condition reduced expression of PKR1 lasts at least 48 h. Note that PKR1 expression was reduced after adipogenic cocktail treatment. Thus, the down regulation of PKR1 remained constant before and after adipogenic induction.

### Analysis of gene expression by quantitative PCR

Total RNA was prepared from 3T3-L1 cells or collected tissues using a Tri-Reagent (MRC, Cincinnati, OH) according to the manufacturer's instructions [29]. Total RNA (2-5 µg) was reverse-transcribed with Super Script II Reverse Transcription Reagents (Invitrogene). The resultant cDNA was subjected to real-time quantitative PCR, in which a specific primers for mouse adiponectin, resistin, TNFα, PPARα, PPARγ, C/EBPα, cyclin D, E, cdk2, c-Myc were used (Table 1). The real-time PCR was carried out in an iCycler myiQ apparatus (Bio-Rad, Life Science Research, Hercules, CA) and SYBR green (Bio-Rad). Relative values of mRNAs were analyzed by the ΔΔC(T) method, normalized to GAPDH mRNA and shown as fold change in expression over control.

### Thermogenesis

Rectal temperature was measured in 40-weeks-old WT and PKR1^−/−^ mice [33]. Mice were caged individually and fasted for 3 h before they were placed in a room maintained in 4°C for cold-exposure experiments. The initial temperature measurements were performed 3 hours before cold exposure (before fasting), at time point 0 (just before cold exposure) and after 2 hours in cold environment.

### Intestinal lipid absorption

WT and PKR1^−/−^ mice were fasted overnight. Mice received 500 µl of olive oil by gavage. Mice were sacrificed 1 hour after gavage feeding and tissues were harvested for histological or oil-red oil analyses [34].

### Fecal fat quantification

Mice were caged individually in metabolic cages with inserts that permit collection and prevent ingestion of fecal material. Mice were maintained for 3 days and feces were collected at days 2 and 3. Fat was extracted from 1 g of feces with 20 ml of chloroform/methanol (2∶1) for 20 min at room temperature as previously described[34].

### Statistic analysis

The results are expressed as means ± SEM. Unless otherwise noted, statistical comparisons for all experiments were performed using Mann–Whitney (for 2 groups) and Kruskal–Wallis (for >2 groups) tests. Statistical comparisons for staining with Oil Red O for adipocytes, CD68 macrophages, PECAM for endothelial cells, Pref-1 for preadipocytes, Tunel and Ki67 for apoptotic nd proliferating cells were performed using the unpaired Student *t* test and ANOVA. *P*<0.05 was considered statistically significant for all tests.

## Results

### PKR1 null mutant (PKR1^−/−^) mice develop obesity

**Figure 1:**
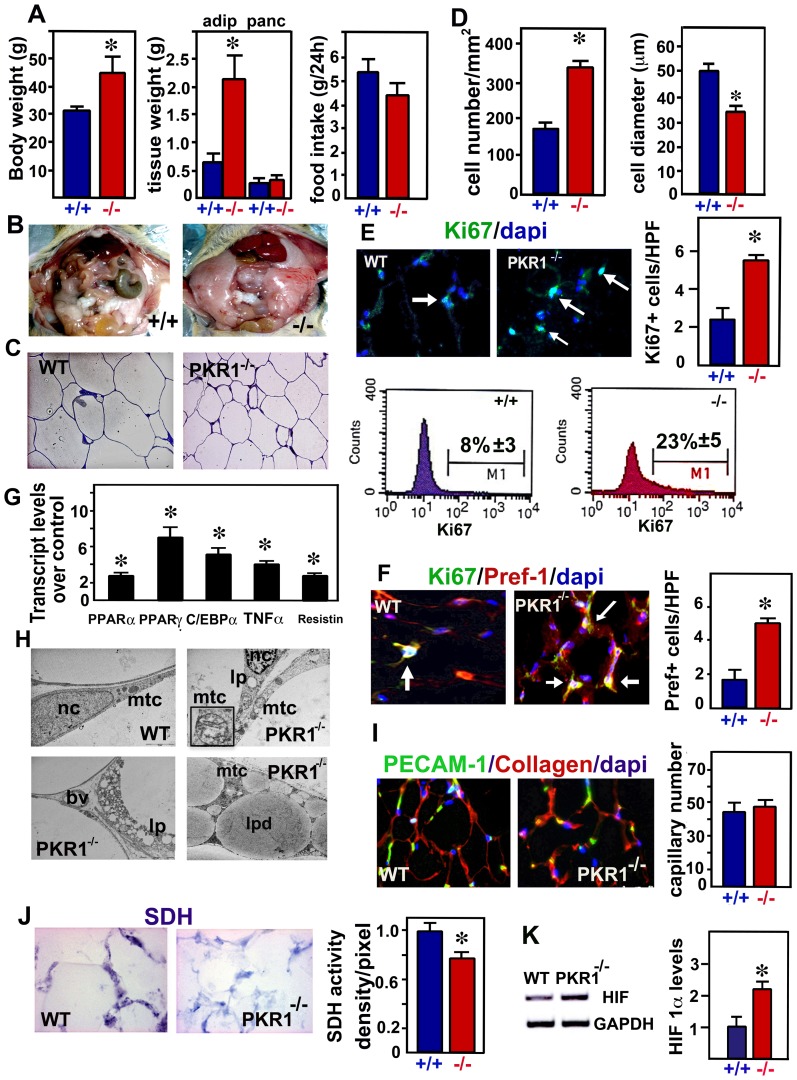
PKR1 null mutant mice (PKR1^−/−^) exhibit obesity at the later age. A) Body weight (g), and WAT and pancreas (pancr) weight (g), and food intake (g/24 h) at the age of 40 weeks (*p<0.05, n = 6). B) Representative illustration of abdominal obesity in PKR1^−/−^ mice. C) Representative illustration of semi-thin sections of adipocyte tissue derived from PKR1^−/−^ and WT mice. D) Histograms show cell number and diameter differences between these two groups of mice. E) Representative illustration of Ki67 positive cells (green) and dapi positive total cells (blue) in adipose tissue of PKR1^−/−^ and WT mice. Quantification of Ki67^+^ cell numbers in the high power field (HPF, ×40 objective) (*p<0.05, n = 20 HPF). Flow cytometery for Ki67 positive nuclei in the cells that were prepared from PKR1^−/−^ and WT type abdominal (epididymal and visceral) WATs tissue after removal of adipocytes. F) Representative illustration and quantification of pref-1^+^/Ki67^+^ positive cells (orange) and dapi positive total cells (blue) in adipose tissue of PKR1^−/−^ and wild type mice (*p<0.05, n = 20 HPF, ×40 objective). G) qPCR analyses of transcript levels of adipogenic genes of PKR1^−/−^ mice over WT mice (fold increase). H) Representative illustration of electron microscopic analyses of adipose tissues derived from PKR1^−/−^ and WT mice demonstrating cytoplasmic lipid droplets (lp) and swollen mitochondria (mtc). Nc: nucleus, bv: blood vessel. I) Illustration and histogram shows the PECAM-1 staining and capillary formation are similar between the adipose tissues of PKR1^−/−^ and WT mice (p<0.05, n = 20 HPF, ×40 objective). J) Illustration and histogram shows the succinyl dehydrogenase (SDH) staining changes between the adipose tissues of PKR1^−/−^ and WT mice (*p<0.05, n = 20 HPF, ×40 objective). K) Western blots analyses reveal a high level of HIF-1α levels in PKR1^−/−^ mice (*p<0.05, n = 3).

PKR1 null mutant (PKR1^−/−^) mice become severely obese on a normal chow diet with body weights ∼40% higher than those of wild-type mice at the age of 40 weeks (Fig. 1A and Fig. S1). However, the mutant and wild-type mice consumed identical amounts of food on the chow diet, ruling out the possibility that PKR1 deficiency results in a greater body weight gain due to hyperphagia (Fig. 1A, right). The epididymal fat pads (subcutaneous and visceral WATs) were also ∼4 times heavier in the PKR1^−/−^ mice than in their wild-type littermates on a normal chow diet (Fig. 1A, middle and Fig. 1B). However, there is no difference in brown adipose tissue mass between two groups of mice. Note that no PKR1 expression was detected in brown tissues. We investigated whether PKR1 deficiency increased WAT mass by the hypertrophy or by hyperplasia of white adipocytes, by measuring the numbers and diameters of WAT in the visceral region of PKR1^−/−^ and wild-type mice. The staining of semi-thin sections revealed that PKR1^−/−^ adipocytes were smaller than wild-type adipocytes, but that their density was higher (Fig. 1C and 1D). However, proliferating Ki67^+^ cells were clearly abundant in PKR1^−/−^ adipose tissue (Fig. 1E and FACS analysis). Most of the Ki67^+^ cells were also positive for the Pref-1 marker of preadipocytes (Fig. 1F). Higher transcript levels for the transcription factors involved in adipogenesis, (PPARα, PPARγ alpha and C/EBPα) and for the markers of inflammation (TNFα) and mature adipocytes (resistin) were observed in PKR1^−/−^ mice, suggesting a possible increase in the conversion of preadipocytes to adipocytes (Fig. 1G). 10% of mature PKR1^−/−^ adipocytes displayed ultra structural features typical of necrosis (i.e., ruptured basal membranes, organelle degeneration), but no detectable features of apoptosis (Fig. 1H). Small cytoplasmic lipid droplets (Fig1H lower panel) and swollen mitochondria that had lost their cristae (Fig1H upper panel) were also observed in the mature PKR1^−/−^ adipocytes. Staining for the angiogenesis marker PECAM-1 revealed no change in the capillary network (Fig. 1I), despite the expansion of adipocyte mass. The mutant mice had lower levels of mitochondrial succinyl dehydrogenase (SDH) activity (Fig. 1J), and higher protein levels of hypoxia-inducible factor-1, a marker of hypoxia (Fig. 1K), clearly demonstrating the occurrence of hypoxia in the mature PKR1^−/−^ adipocytes with defective capillary formation.

### PKR1 null mutant mice displayed diabetes-like disorders

**Figure 2:**
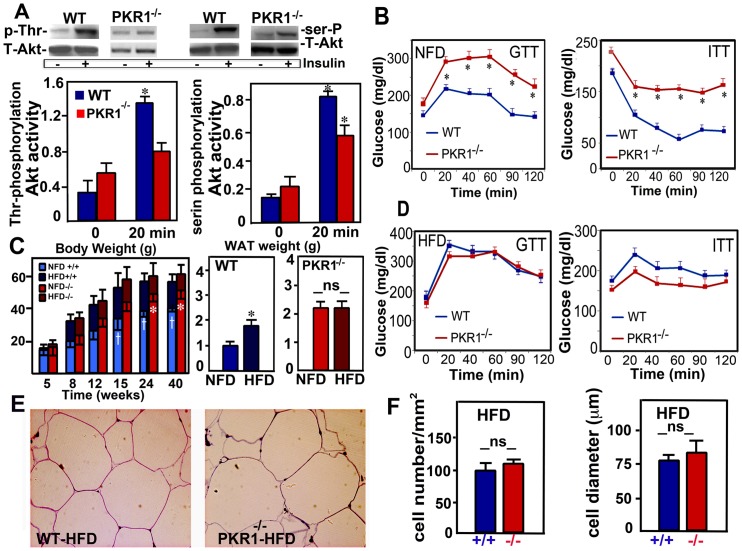
PKR1 null mutant mice exhibit a diabetes-like syndrome. A) Western blot analyses on lysates derived from the adipose tissue of both group of mice 0 or 20 min after i.p. 0.75U/I insulin injections. Western blot analyses revealed that insulin-stimulated phosphorylation of Akt (at serine and threonine) were significantly lower in PKR1^−/−^ adipose tissue as compare to wild (WT) adipose tissue (* p<0.05, n = 3). B) Glucose tolerance (GTT) and Insulin tolerance tests (ITT) in 40 weeks old mice. PKR1^−/−^ mice did not tolerate glucose load and displayed impaired ITT (* p<0.05, n = 8). C) Body weight increases in HFD-fed and NFD-fed mice. * shows a significant difference between NFD-fed PKR1^−/−^ mice and WT mice, n = 8. t shows difference between HFD-fed and NFD-fed WT mice (blue). D) GTT and ITT in HFD-fed mice at the age of 40 weeks. No significant alteration was observed between the groups, due to severe increase in GTT and ITT of WT mice treated with HFD (* p<0.05, n = 8). E) Representative illustration of semi-thin sections of adipocyte tissue derived from PKR1^−/−^ and WT mice after HFD feeding showing the hypertrophic adipocytes in both groups (×40). F) Histogram shows cell numbers and diameters are similar between these two groups of mice (ns = no significance, p>0.05, n = 20).

We then investigated possible impairment of the insulin response in hypoxic PKR1^−/−^ WAT tissues. We found that the insulin-stimulated phosphorylation of Akt kinase at the threonine 308 and serine 478 residues was severely attenuated in PKR1^−/−^ WAT tissues (Fig. 2A left). We analyzed whether PKR1-deficient mice displayed changes to glucose homeostasis and metabolism. Glucose and insulin tolerance tests (ITT) showed that these animals were significantly less able to handle glucose loading than wild-type mice, at the age of 40 weeks. In the glucose tolerance test (GTT), PKR1-deficient mice displayed impaired glucose tolerance, with higher blood glucose concentrations 20, 40, 60, 90 and 120 min after glucose administration than were recorded for wild-type mice (Fig. 2B left). Insulin sensitivity was also significantly lower in PKR1-null mutant mice than in wild-type mice (Fig. 2B right). An impaired glucose tolerance was also observed in PKR1^−/−^ mice at the age of 15 weeks (Fig. S2). No significant differences in food intake and net energy expenditure (thermogenesis) (Fig. S6A) and changes in the expression of energy metabolism-related genes in the skeletal muscle (Fig. S6B). Jejunum of PKR1^−/−^ mice displayed no significantly higher levels of lipid absorption one hour after gavage with oil (Fig. S6C). The levels of lipid excretion in the feces did not alter for PKR1^−/−^ mice and wild-type mice on the chow diet (Fig. S6D), suggesting that PKR1^−/−^ mice did not show abnormal absorption of calories.

Next we investigated the effect of a dietary fat composition on the obese phenotype, by feeding the wild type and PKR1^−/−^ mutant mice a high-fat diet. The high-fat diet greatly decreased the differences in body weight between the two groups, due to a massive increase in the body weight of wild-type mice (Fig. 2C). The PKR1^−/−^ and wild-type mice ingested identical amounts of food on the high-fat diet (2.56±0.35 and 2.9±0.39 g/day, respectively). Consistent with these findings, mutant and wild-type mice had similar GTT and ITT results 34 weeks after high-fat diet treatments, due to a diabetes-like increase in glucose and insulin tolerance in the wild-type mice (Fig. 2D). Both groups exhibited hypertrophic visceral adipocytes (Fig. 2E) without alterations in cell number and diameter (Fig. 2F).

### Adipocyte specific loss of PKR1 in mice exhibit enhanced abdominal adiposity

**Figure 3:**
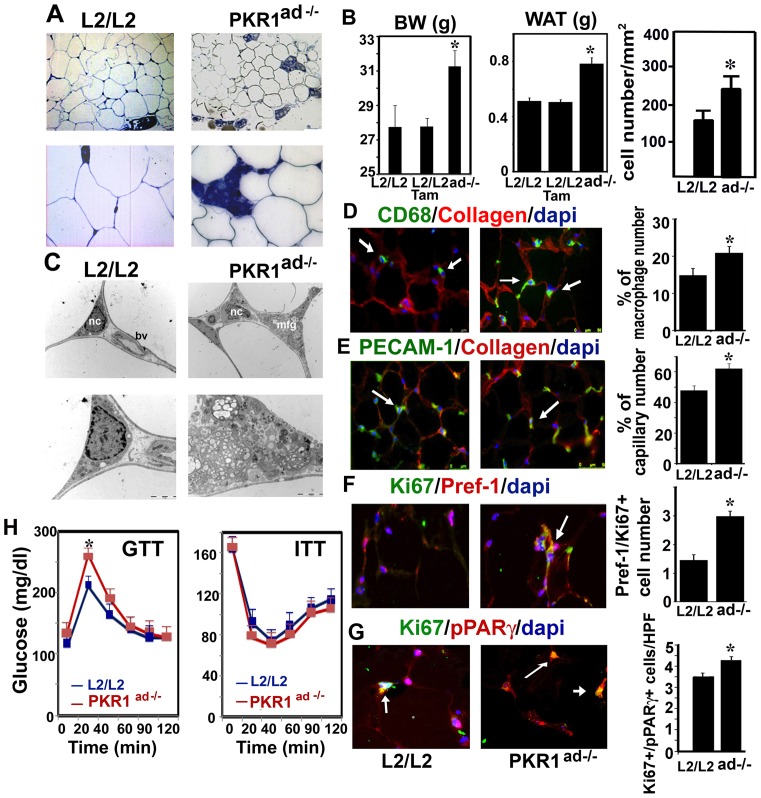
Abdominal obesity in PKR1^ad−/−^ mice is due to preadipocyte proliferation and differentiation. A) Representative illustration of semi thin analyses of adipose tissues derived from control (L2/L2) and PKR1^ad−/−^ mice. Blue shows macrophage accumulation. B) Body (g) and WAT weight (g), and adipocyte number of the PKR1^ad−/−^ and L2/L2 mice (*p<0.05, n = 6). C) Representative illustration of electron microscopic analyses of adipose tissues derived from PKR1^ad−/−^ and L2/L2 mice, showing macrophage deposition in PKR1^ad−/−^ mice. D) Macrophage specific CD68 staining and quantification of macrophages on cryosectioned adipose tissues derived from PKR1^ad−/−^ and L2/L2 mice (*p<0.05, n = 20 HPF, ×40 objective). E) Illustration and histogram shows the PECAM-1 staining and capillary formation changes between the adipose tissues of PKR1^ad−/−^ and L2/L2 mice (*p<0.05, n = 20 HPF, ×40 objective). F) Representative illustration and quantification of pref-1^+^/Ki67^+^ positive cells (orange) and dapi positive total cells (blue) in adipose tissue of PKR1^ad−/−^ and L2/L2 mice (*p<0.05, n = 20, HPF, ×40 objective). G) Representative illustration and quantification of PPARγ^+^/Ki67^+^ positive cells (orange) and PPARγ/dapi positive total cells (violet) in adipose tissue of PKR1^ad−/−^ and L2/L2 mice (*p<0.05, n = 20 HPF, ×40 objective). H) GTT and ITT in 24 weeks old mice (*p<0.05, n = 6).

To eliminate an endothelial-adipocyte interaction and possible central regulation of the body weight in mutant mice, we generate tamoxifen-inducible adipocyte/preadipocyte-specific PKR1 knockout mice (Fig. S3A, B and C). PKR1^ad−/−^ mice had a 13±2% higher body weight (Fig. 3B left) and 1.5 times higher epididymal mass (Fig. 3B middle) and an increased number of adipocytes at the age of 24 weeks (Fig. 3A and 3B right). The PKR1^ad−/−^ and L2/L2 mice ingested similar amounts of food (4.56±0.35 and 4.9±0.39 g/day, respectively). Semi-thin sections and electron microscopy showed interstitial macrophage deposition in PKR1^ad−/−^ adipose tissue, which was confirmed by macrophage-specific, CD68 staining (Fig. 3A, C and D). Capillary formation, as detected by PECAM-1 staining, was also elevated in PKR1^ad−/−^ adipose tissues (Fig. 3E). The PKR1^ad−/−^ adipose tissues contained a 3 times higher number of Ki67^+^/Pref-1^+^ cells (Fig. 3F). We then determined the number of newly generated adipocytes by double-immunostaining for Ki67 and PPARγ. Almost 14% of the Ki67^+^ cells also expressed PPARγ (Fig. 3G), indicating that the adipocytes concerned had recently been generated by preadipocyte proliferation in the PKR1^ad−/−^ adipose tissues. (The specificities of antibodies for pref-1 and PPARγ were tested on adipose tissues and shown in Fig. S3D). Thus, adipose tissue plasticity in PKR1^ad−/−^ mice induces higher WAT mass by increasing preadipocyte proliferation and conversion to adipocytes. Albeit the initial glucose levels were higher in 24 week-old mice PKR1^ad−/−^ mice, the glucose clearance was similar to control group in response to glucose loading at the age of both 24 (Fig. 3H) and 40 weeks (Fig. S4). The ITT remained similar in both groups at the both 24 and 40 week-old ages (Fig. 3H and Fig. S4).

### Prokineticin-2 inhibits proliferation of 3T3-L1 cells

**Figure 4:**
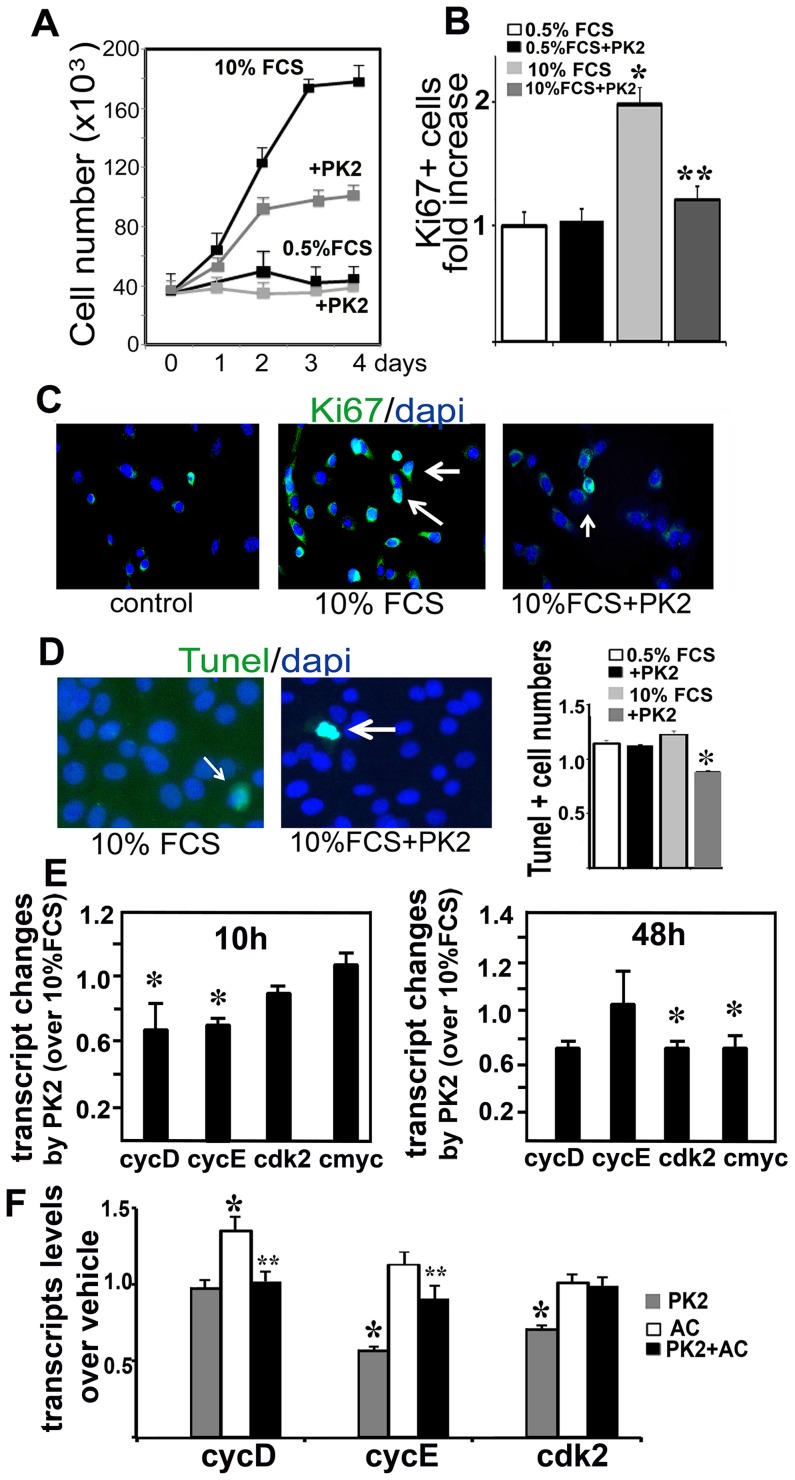
Prokineticin-2 inhibits proliferation of 3T3-L1 cells. A) 10% FCS increased cell numbers within 24 h as compare to cell numbers in 0.5% serum treated cells reaching to maximum within 4 days. Treatment of the cells with prokineticin-2 (5 nM) significantly reduced cell numbers as compare to that of 10% FCS alone. B) Ki67 positive proliferating cells in S phase were increased approximately 2 times with 10% FCS treatment of the 3T3-L1 cells (**p<0.05, n = 20). Pretreatment of the cells with prokineticin-2 completely abolished Ki67 positive cell number (*p<0.05, n = 4). C) Representative illustration of Ki67 positive cells (green) and dapi positive total cells (blue) in control (0.5%FCS), 10%FCS and PK2+10%FCS groups (HPF, ×40 objective). D) Representative illustration of Tunel positive cells (green) and dapi positive total cells (blue) in each group. No detectible apoptotic cells were observed in either group (n = 20, HPF, ×40 objective). E) qPCR analyses for cycling gene expression 10 hours (left) and 48 hours after 10% FCS and 10% FCS+Prokineticin-2 treatment. Transcript of Cyclin D and E levels (fold increase) were reduced 10 h after treatment of the prokineticin-2, whereas cdk2 and c-Myc were reduced 48 h after prokineticin-2 treatment (*p<0.05, n = 4) as compare to that of 10% FCS alone. F) Histogram shows quantification of transcript levels for cyclin E, D and cdk2 48 after adipogenic cocktail (AC) treatment following prokineticin pretreatment for 10 hours in 3T3-L1 cells as compare to that of vehicle (n = 5, * different from vehicle, ** different from AC, p<0.05).

To gain further insights into the functional interactions governing adipose tissue expandability, we investigated whether PKR1 has a cell-autonomous role in preadipocytes 3T3-L1. For control 3T3-L1 cells, the number of cells began to increase about 24 h after induction with 10% FCS. However, there were fewer cells 24–72 h after induction following prokineticin-2 (5 nM or 10 nM) treatment than after treatment with 10% FCS alone (Fig. 4A). We then investigated the cell cycle by Ki67 staining (Fig 4B and 4C). Entry into S phase increased within 10 h, in cells grown in 10% FBS but not in cells grown in 0.5% FCS. However, the increase in the number of Ki67^+^ cells induced by 10% FCS was inhibited by prokineticin-2 treatment. No differences were observed between prokineticin-2-treated and control cells in culture medium supplemented with 0.5% FCS, indicating that prokineticin-2 had no cytotoxic effect (Fig. 4B). A direct apoptotic effect of prokineticin-2 was excluded by TUNEL staining, which revealed no detectable apoptosis 48 hours after treatment of the cells with prokineticin-2 (Fig. 4D). These data suggested that 3T3-L1 cells were at a similar degree of confluence and that there was no cytotoxicity or apoptosis induction in response to prokineticin-2 treatment. Strikingly, prokineticin-2 induced a marked decrease in the expression of molecular markers of the cell cycle, such as cyclin E, cyclin D (within 10 hours), Cdk2 and c-Myc (within 48 hours) as compare to 10% FCS alone (Fig. 4E). Pretreatment of the 3T3-L1 cells with prokineticin-2 also downregulated the cell cycle gene expression 48 hours after the administration of an adipogenic cocktail (insulin, dexamethasone and isobutylmethylxanthine) (Fig. 4F). Prokineticin-2 mediated this effect was completely abolished after acute PKR1 knockdown, achieved through the use of an siRNA targeting PKR1. From day 48, PKR1 mRNA levels were 70% lower after transfection with PKR1 siRNA than after transfection with a nonspecific siRNA (Fig. S5A and B).

### Prokineticin-2 inhibits adipogenic differentiation of preadipocytes

**Figure 5:**
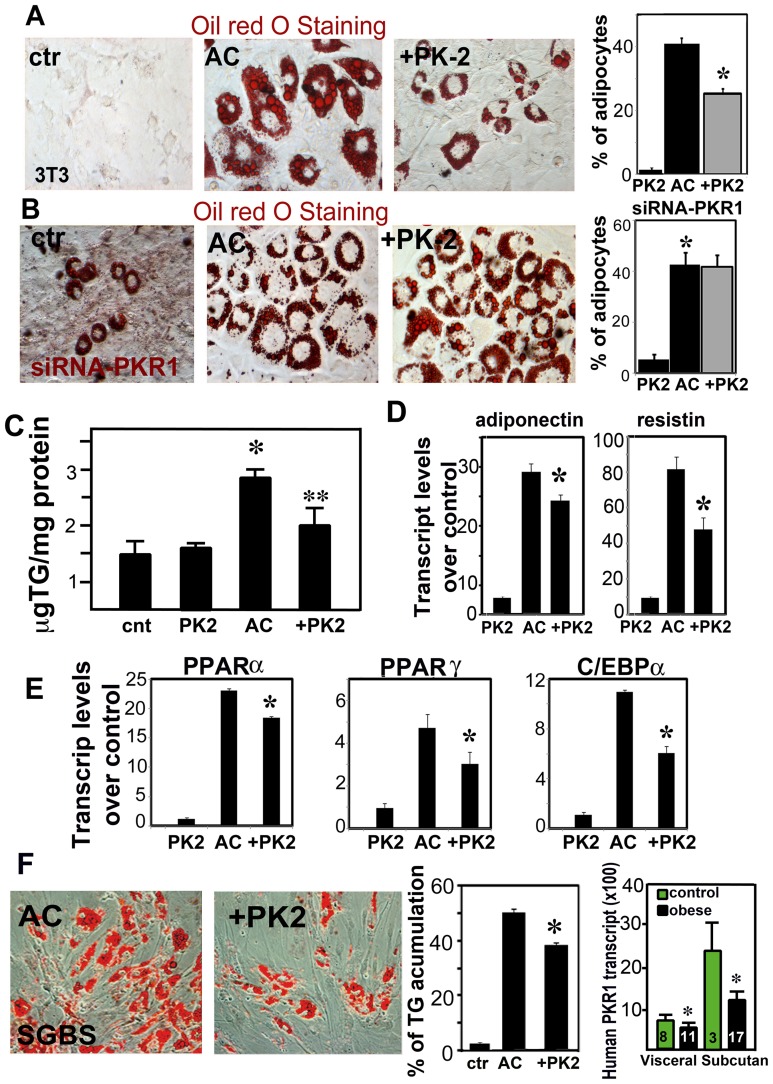
Prokineticin-2 inhibits preadipocyte conversion to adipocytes. A) Oil-red O staining of 3T3-L1 cells treated with control (ctr), adipogenic cocktail (AC) for 8 days or pretreated with 5 nM of prokineticin-2 (+PK-2) 10 h before AC treatment (n = 20, *p<0.05, HPF, ×40 objective). B) In the PKR1 knockdown 3T3-L1 cells (siRNA-PKR1), prokineticin-2 (5 nM) pretreatment did not able to inhibit adipogenesis induced by AC. Histogram shows pretreatment of adipocytes with prokineticin-2 reduced number of adipocytes detected by Oil red staining in the control siRNA transfected 3T3-L1 cells but not in the PKR1 siRNA transfected cells (n = 20, *p<0.05, HPF, ×40 objective). C) Triglyceride (TG) accumulation in the cell lysates. * shows PK2 significantly diminished AC effect (n = 3, p<0.05). D) qPCR analyses show increased levels of mature adipocyte markers, resistin and adiponectin by AC that was inhibited by prokineticin-2 as compare to that of AC (n = 3, p<0.05). E) qPCR analyses reveal that PPARα, PPARγ and C/EBPα, were significantly reduced by prokineticin-2 pretreatment as compare to that of AC (n = 3, p<0.05). F) Representative illustration and histogram shows that prokineticin-2 reduced human preadipocyte SGBS cell differentiation into adipocytes and TG accumulation induced by AC (n = 3, p<0.05). Quantitative PCR analyses show that transcript levels of human PKR1 (right panel) were reduced in obese visceral and subcutaneous human tissues (n is indicated in the figure, *p<0.05).

To next explore the *in vitro* role of prokineticin 2 in conversion of preadipocyte to adipocyte, the 3T3-L1 cells were grown to confluence and their differentiation into adipocytes was induced with an adipogenic cocktail, following treatment of the cells with prokineticin-2 or vehicle. The prior treatment of the 3T3-L1 cells with prokineticin-2 (5 nM) induced a significant decrease in adipogenic cocktail-mediated lipid accumulation (Fig. 5A and histogram) and TG accumulation (Fig. 5C). We evaluated the consequences of PKR1 depletion for preadipocyte differentiation further, by studying the effects of prokineticin-2 on 3T3-L1 adipogenesis after acute PKR1 knockdown, achieved through the use of an siRNA targeting PKR1 (Fig. S5A). In these conditions, spontaneous adipocyte formation was observed in 3T3-L1 cells transfected with an siRNA targeting PKR1. The prokineticin-2-mediated inhibition of adipogenesis was completely abolished, suggesting a role for PKR1 in this process (Fig. 5B and histogram). Accordingly, in the presence of PKR1, prokineticin-2 induced a marked decrease in the expression of molecular markers of mature adipocytes, such as adiponectin and resistin (Fig. 5D) and significantly decreased mRNA levels for PPARα, PPARγ alpha and C/EBPα (Fig. 5E). Prokineticin-2 also inhibits human preadipocyte cell strain SGBS conversion to adipocyte 10 days after adipogenic induction (Fig. 5F left and middle panel). Interestingly, transcript levels of human PKR1 (right panel) were significantly reduced in obese visceral and subcutaneous human tissues. Together, these *in vitro* data provide a convincing demonstration that PKR1 plays a key role in suppressing preadipocyte conversion to adipocytes.

## Discussion

The identification of factors involved in white fat tissue growth is important to elucidate the etiology of obesity. Albeit recent studies have provided insights into the anorexigenic and angiogenic properties of prokineticin-2, its physiological functions in preadipocytes and adipocytes are unknown. In this study, we provide *in vivo* and *in vitro* evidence for distinct roles of prokineticin receptor-1 (PKR1) in maintenance of proliferation and conversion of preadipocytes to adipocytes, controlling abdominal adipocyte numbers and adipose tissue mass.

Both PKR1^−*/*−^ and PKR1^ad−/−^ mice displayed abdominal obesity, but only PKR1^−*/*−^ mice had peripheral obesity accompanied by a diabetes-like syndrome, suggesting non-adipocyte PKR1-mediated events also contribute to development of peripheral obesity with diabetes-like syndrome. Angiogenesis and adipogenesis interact reciprocally via paracrine signaling systems in the development of adipose tissue [35]. In PKR1^−/−^ mutants, the development of the new capillaries was obstructed due to loss of angiogenic PKR1 in endothelial cells as well. Therefore, the reciprocal regulation of adipogenesis via angiogenesis is impaired in the PKR1^−/−^ mice, creating a hypoxic environment. High levels of HIF1α, an indicator of hypoxic stresses, in PKR1^−/−^ adipocytes may also contribute to impaired adipocyte insulin signaling [36]. Consistently, PKR1^−/−^ WATs displayed impaired Akt phosphorylation in response to insulin. PKR1 deficient mice were obese and had poorer GTT and ITT responses than wild-type mice. In contrast to PKR1^−/−^ mice, PKR1^ad−/−^ mice had higher levels of capillary formation in fat tissues, indicating, the reciprocal regulation of adipogenesis via angiogenesis is not impaired in the PKR1^ad−/−^ mice. Moreover, glucose and insulin tolerance remained unaffected in the PKR1^ad−/−^ mice, indicating that a damaged endothelial-adipocyte interaction may involve in the impaired insulin response in PKR1-null mutant adipocytes. The obese phenotype of PKR1^−/−^ mice did not seem to be modified by dietary fat content, because the high-fat diet resulted in smaller differences in body weight and GTT and ITT responses, due to the massive increase in body weight observed in wild-type mice. However, high-fat diet promoted hypertrophy in the both PKR1^−/−^ and wild adipocytes, indicating that high calorie intake is the key factor in conversion of hyperplasia to hypertrophy in PKR1^−/−^ adipocytes.

It seems unlikely that the hypothalamic control of energy metabolism is involved in the adipocyte hyperplasia observed in PKR1-null mice. PKR2, which is strongly expressed in the hypothalamus, is responsible for the central anorexic and thermoregulatory effect of prokineticin-2 [10]. Chronic continuous infusion of PK2 via the brainstem significantly reduced body weight and food intake in a mouse model of human obesity without altering energy expenditure [37]. Central regulation of the body weight was eliminated in our PKR1 ^ad-/-^ mice that also had an increase in abdominal fat mass accumulation, due to accelerated preadipocyte proliferation promoting the formation of new adipocytes. Accordingly, preadipocyte hyperplesia in the adipose tissue [38] exacerbates the accumulation of white adipocytes, and is also a key event in the development of some types of obesity [39]. In this study, we also discovered that PKR1 also has essential functions in controlling preadipocyte proliferation largely through the downregulation of cell cycle genes (cyclin D, E, cdk2, c-Myc). Adipocytes exhibit continual turnover in adult humans [5]. Increases in adipocyte number during aging have been implicated in the severity of obesity in the elderly [40]. The undifferentiated adipocyte precursors, preadipocytes residing in adipose tissue vascular stroma are capable of proliferating and differentiating into an adipose deposit in response to obesogenic triggers [41]. Recently loss of necdin in mice has been shown to promote a hyperplasic adipocyte due to increase in preadipocyte proliferation and differentiation [42]. Postconfluent DNA replication, cell division and cell proliferation during mitotic clonal expansion are required for initiation of the transcriptional cascade for preadipocyte differentiation [43]. Prokineticin-2 inhibited not only mouse embryonic preadipocyte but also adult human preadipocyte conversion to adipocyte. We also reported here that the expression of prokineticin-2 (Fig. S7) and its receptor PKR1 was altered in human WAT tissues. Possible mutations on these genes in human obesity remain to be determined.

A number of angiogenic factors, including epidermal growth factor, platelet-derived growth factor BB [44], basic fibroblast growth factor [45] and heparin-binding epidermal growth factor-like growth factor (HB-EGF) [46], inhibit the conversion of preadipocytes to adipocytes [47], much like we find with prokineticin-2.

Conclusively, this novel function of PKR1 as a major suppressor of preadipocyte proliferation and conversion to adipocytes will expand our knowledge on factors regulating adipocyte expansion in the alarming world-wide trend toward increasing obesity and its associated pathologies. Preadipocyte replication represents a new mechanism for expansion of fat mass in cases of human obesity and reducing preadipocyte replication may represent a new avenue of therapeutic intervention against obesity [48]. As 40% of drugs target GPCRs [49]PKR1 could be an effective target for treatment or prevention of obesity.

Study Limitations: Recent studies suggest preadipocytes can differentiate to macrophages, dedifferentiate from adipocytes or macrophages, and differentiate back to adipocytes [50]. Since *aP2-Cre*ERT2 line has been found to induce recombination in the adipose tissue, capillary endothelium in the heart and in intermyofibrillar cells in the skeletal muscle, but not in macrophages in adipose tissue [51], thus we can exclude effect of macrophage on the observed phenotype of PKR1^ad−/−^ mice. Our data show that the reciprocal regulation of adipogenesis via angiogenesis is not impaired in the PKR1^ad−/−^ mice, eliminating possible contribution of endothelial cells in the PKR1^ad−/−^ mice. The contribution of the endothelial-PKR1 signaling on regulation of metabolic homeostasis remains to be investigated.

## Supporting Information

Figure S1 **40 weeks old PKR1^−/−^ null mutant mice exhibit hypoxic adipocytes.** Representative illustration showing increased body weight of PKR1^−/−^ mice (left) compare to wild type (right).
(TIF)

Figure S2 **Metabolic changes on PKR1^−/−^ null mutant mice at the 15 week-old age.** GTT test shows PKR1-deficient mice have abnormal glucose clearance, beginning at 40 min postglucose treatment as compare to age matched wild type mice (n = 8).
(TIF)

Figure S3 **Generation of PKR1^ad−/−^ mice and Immunostaings on adipose tissues.** **A)** Representative genotype analysis of PKR1^ad−/−^ mice. Genomic DNA was amplified with oligonucleotide primers detecting aP2-Cre and PKR1^lox/lox^ alleles. PKR1^ad−/−^mice harbor the aP2-Cre transgene and are homozygous for the PKR1 floxed allele (PKR1^lox/lox^). Control mice (L2/L2) are aP2-Cre negative and they are PKR1^lox/+^. B) RT-PCR analyses on RNAs extracted from adipose tissue revealed that PKR1^ad−/−^ mice had lower Pkr1levels after tamoxifen treatment. C) Representative illustration of loss of PKR1 protein in the adipose tissue of PKR1^ad−/−^ mice by immunostaining of the cryosectioned adipose tissue with PKR1 antibody. D) Representative illustration of immunostatining of PKR1^ad−/−^adipocytes with dapi, Ki67 and pref-1 antibodies and corresponding secondary antibodies (upper). Pref-1 and PPARγ antibody stainings of the adipose tissues without secondary antibodies show no non-specific stainings of the adipose tissues with the first antibodies.
(TIF)

Figure S4 **Glucose clearance after glucose and insulin treatment of 40 weeks old PKR1^ad−/−^ mutant mice.** GTT test showed that glucose clearance in PKR1^ad−/−^ mice was similar to control group in response to glucose loading at the age of 40 weeks (left). The ITT remained similar in both groups at the 40 week-old ages (n = 6, p>0.05) (right).
(TIF)

Figure S5 **Prokineticin-2 mediated proliferation rate in 3T3-L1 cells, expressing low level of PKR1. A)** Representative illustration of PCR analyses revealed that siRNA for PKR1 significantly reduced PKR1 expression 48 hours after siRNAPKR1 transfection. Histogram shows quantification of the PKR1 expression levels in each group (n = 3, *p<0.05). siRNA NS: nonspecific siRNA, siRNA PKR1: siRNA for PKR1. B) PK-2 was not able to inhibit proliferation induced by 10% FCS for 3 days in the 3T3-L1 cells transfected with siRNA for PKR1 (n = 3, *p<0.05 different then initial 0 time). In the 3T3-L1 cells transfected with siRNA-NS, PK-2 inhibits % FCS induced proliferation rate (*p<0.05 different then initial 0 time ** p<0.05 different than 10% serum).
(TIF)

Figure S6 **Thermogenesis, expression profiles of energy metabolism-related genes in the muscle and lipid absorption in PKR1^−/−^ null mutant mice.** A) Body temperature of PKR1-deficient (PKR1^−/−^) and wild (WT) mice (n = 6), 3 hours before cold exposure (room temperature), the time of cold exposure (0) and 2 hours after cold (4°C) exposure. B) Total RNA was extracted from the muscle of 40-week-old mice. qRT-PCR was performed using primers listed in Table 1. The values in the muscle were normalized with those of β-actin. No statistically significant differences were noted between WT^+/+^ and PKR1^−/−^ mice (p>0.05, n = 3). PPARs, peroxisome proliferators-activated receptor; GLUT4, glucose transporter type 4; UCP, uncoupling protein. All data are presented as mean ± SEM (*n* = 4). C) Representative of semi-thin analyses of Jejunum derived from mutant and wild type mice 20 min after oil (vegetable oil) gavages indicating slightly higher levels of lipid absorption in the jejunum of PKR1^−/−^ mice one hour after gavage with oil. C) Quantification of intestinal lipid levels after oil-red staining revealed a similar oil-red staining between the groups. D) The intestinal lipid/feces lipid ratio was not significantly altered between the groups (n = 6, p<0.05).
(TIF)

Figure S7 **Prokineticin-2 and its receptor PKR1 expression in Human adipose tissues.** Quantitative PCR analyses show transcript levels of prokineticin-2, a ligand were increased in obese human WAT tissues.
(TIF)
