# Supplementary figures and images for: Prokineticin Receptor 1 as a Novel Suppressor of Preadipocyte Proliferation and Differentiation to Control Obesity
Source: PLoS One. 2013 Dec 4;8(12):e81175. doi: 10.1371/journal.pone.0081175 (PMC3852222; doi:10.1371/journal.pone.0081175)

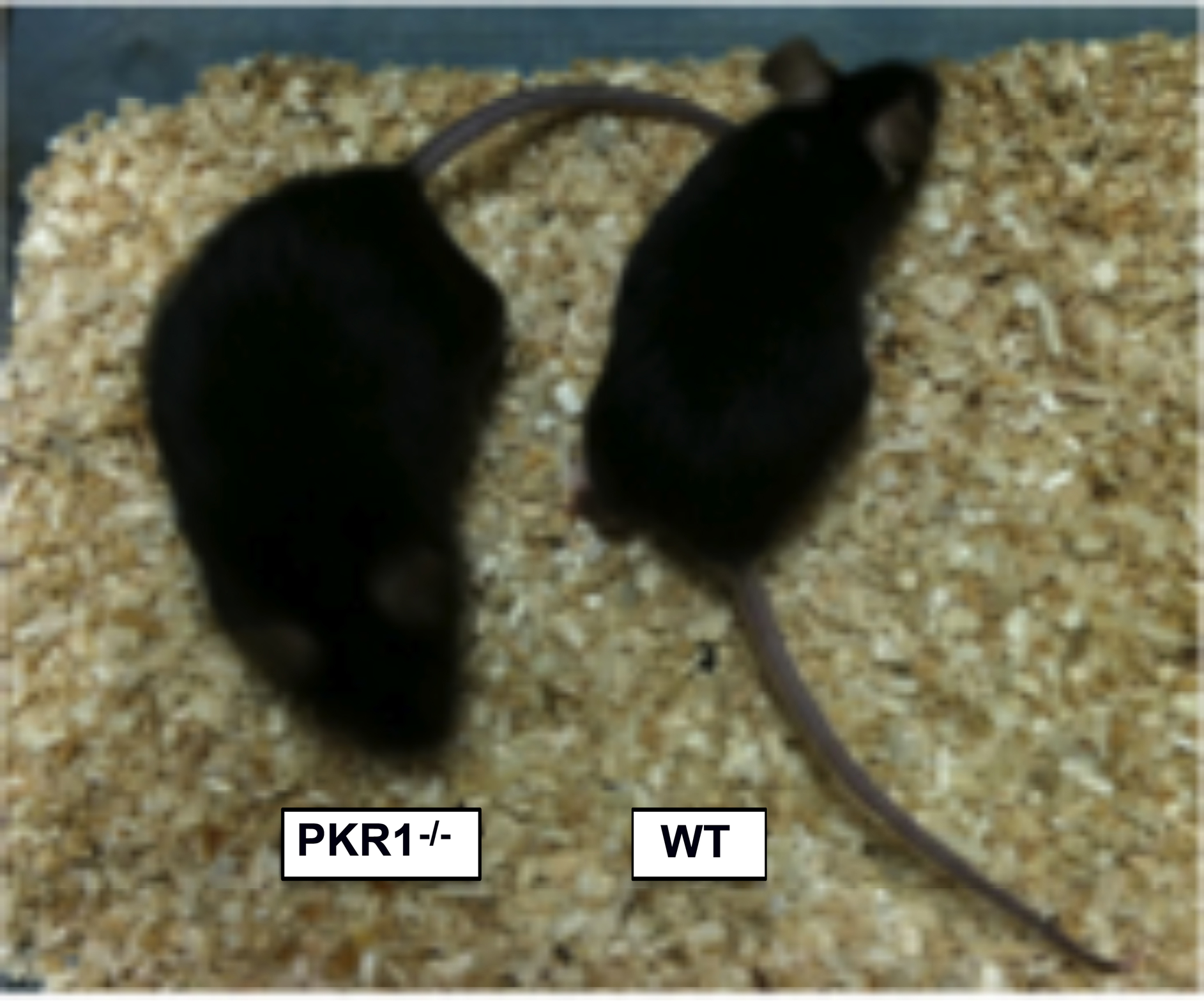

Supplement: Figure S1 — 40 weeks old PKR1−/− null mutant mice exhibit hypoxic adipocytes. Representative illustration showing increased body weight of PKR1−/− mice (left) compare to wild type (right). (TIF) [file pone.0081175.s001.tif]

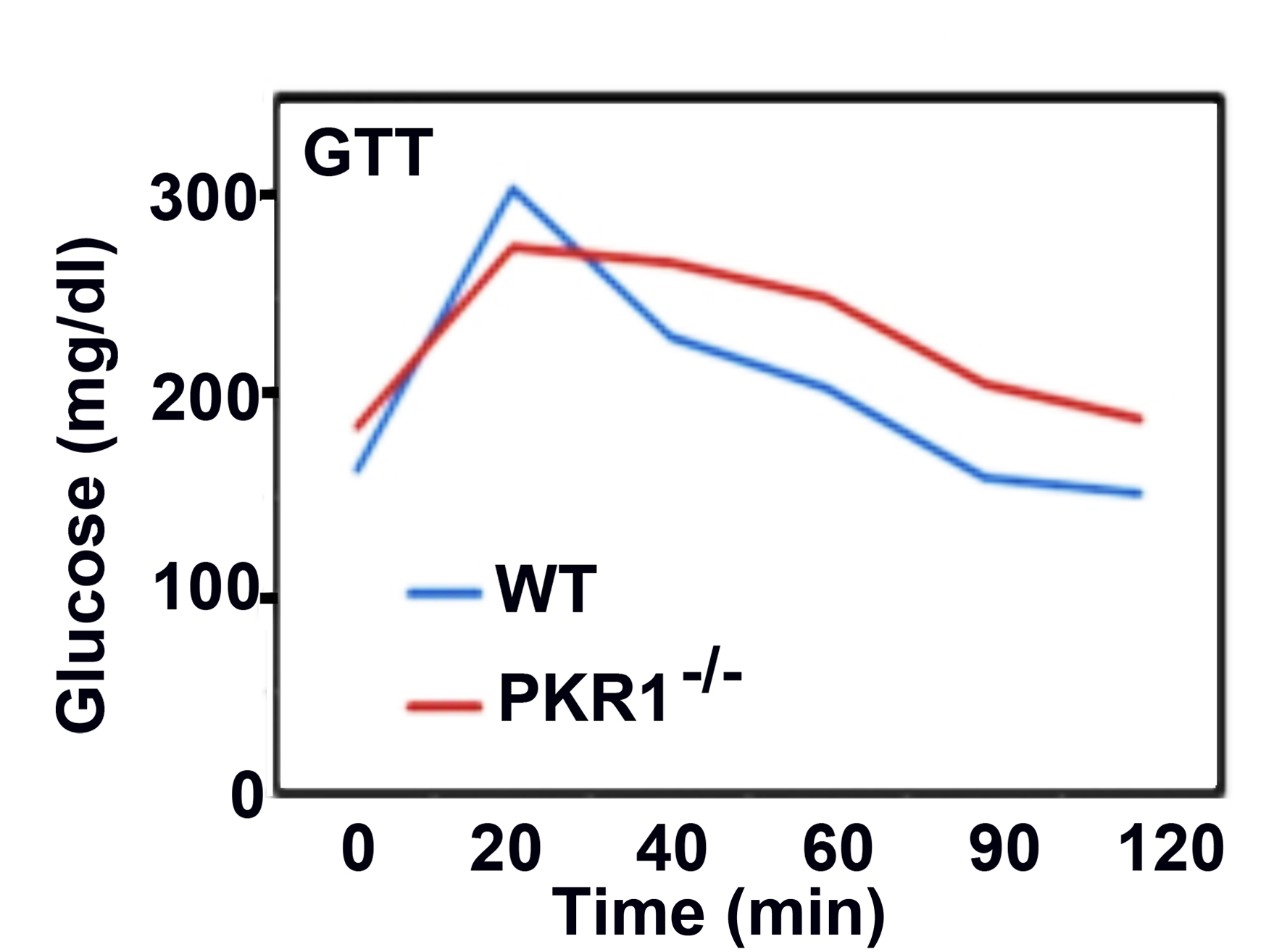

Supplement: Figure S2 — Metabolic changes on PKR1−/− null mutant mice at the 15 week-old age. GTT test shows PKR1-deficient mice have abnormal glucose clearance, beginning at 40 min postglucose treatment as compare to age matched wild type mice (n = 8). (TIF) [file pone.0081175.s002.tif]

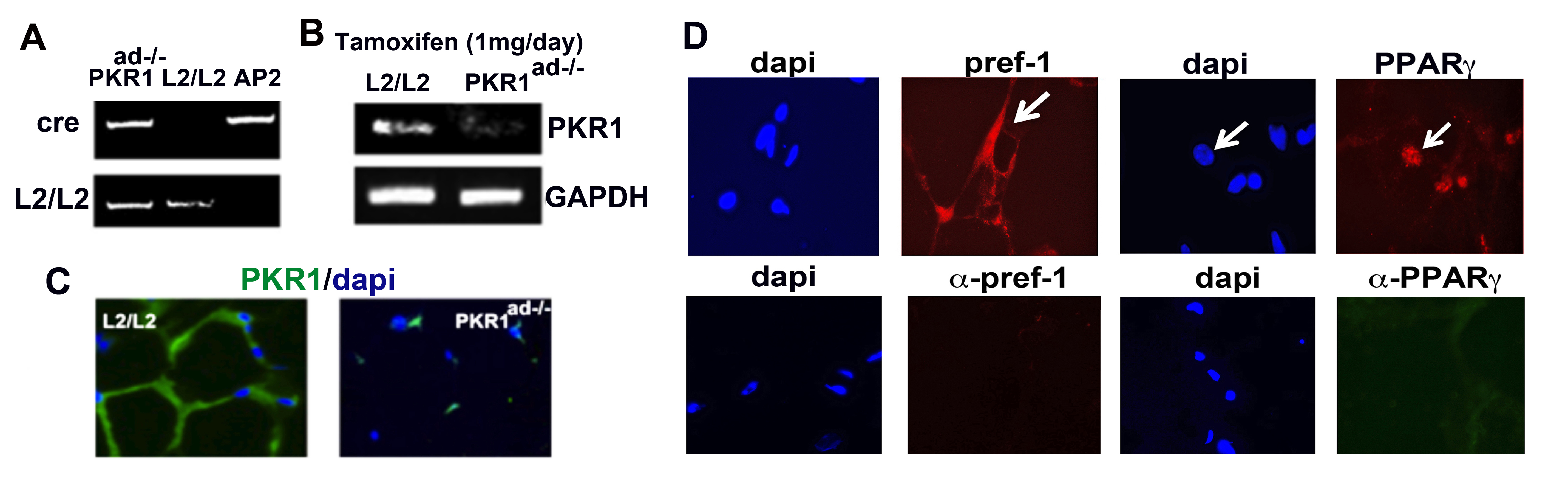

Supplement: Figure S3 — Generation of PKR1ad−/− mice and Immunostaings on adipose tissues. A) Representative genotype analysis of PKR1ad−/− mice. Genomic DNA was amplified with oligonucleotide primers detecting aP2-Cre and PKR1lox/lox alleles. PKR1ad−/−mice harbor the aP2-Cre transgene and are homozygous for the PKR1 floxed allele (PKR1lox/lox). Control mice (L2/L2) are aP2-Cre negative and they are PKR1lox/+. B) RT-PCR analyses on RNAs extracted from adipose tissue revealed that PKR1ad−/− mice had lower Pkr1levels after tamoxifen treatment. C) Representative illustration of loss of PKR1 protein in the adipose tissue of PKR1ad−/− mice by immunostaining of the cryosectioned adipose tissue with PKR1 antibody. D) Representative illustration of immunostatining of PKR1ad−/−adipocytes with dapi, Ki67 and pref-1 antibodies and corresponding secondary antibodies (upper). Pref-1 and PPARγ antibody stainings of the adipose tissues without secondary antibodies show no non-specific stainings of the adipose tissues with the first antibodies. (TIF) [file pone.0081175.s003.tif]

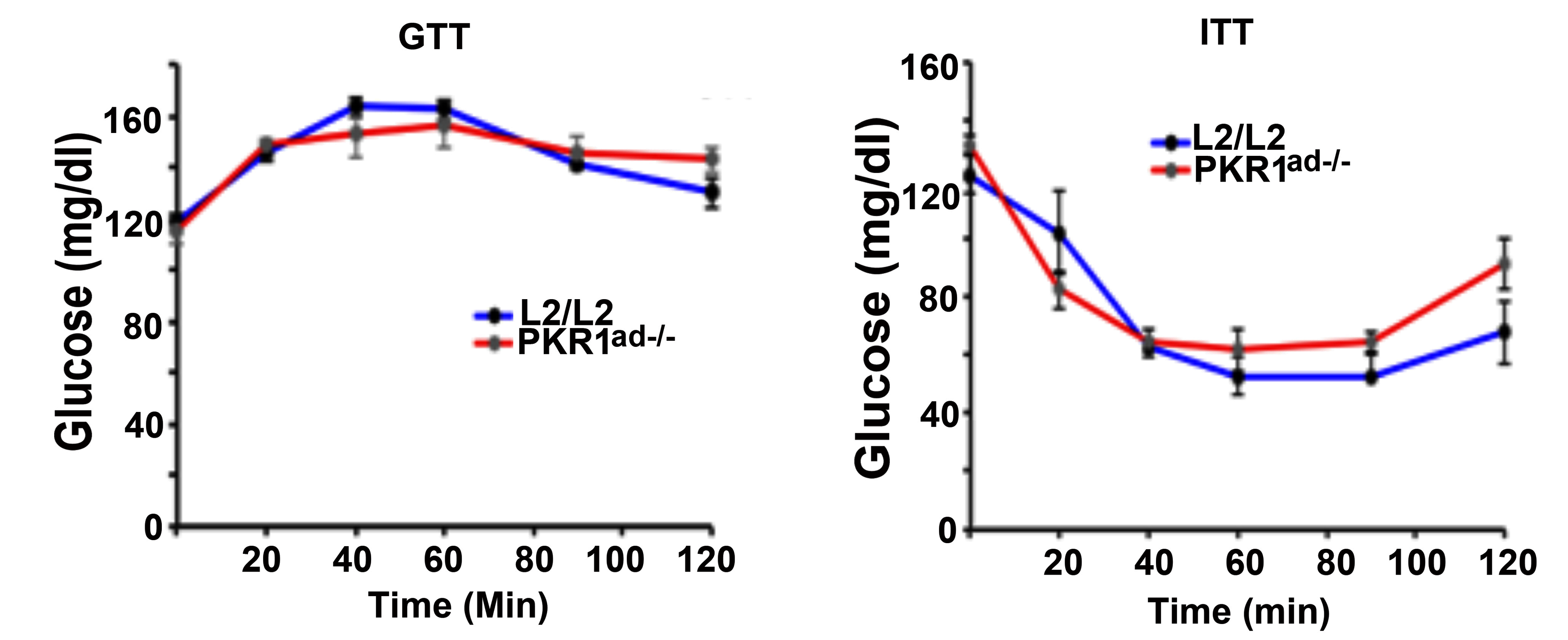

Supplement: Figure S4 — Glucose clearance after glucose and insulin treatment of 40 weeks old PKR1ad−/− mutant mice. GTT test showed that glucose clearance in PKR1ad−/− mice was similar to control group in response to glucose loading at the age of 40 weeks (left). The ITT remained similar in both groups at the 40 week-old ages (n = 6, p>0.05) (right). (TIF) [file pone.0081175.s004.tif]

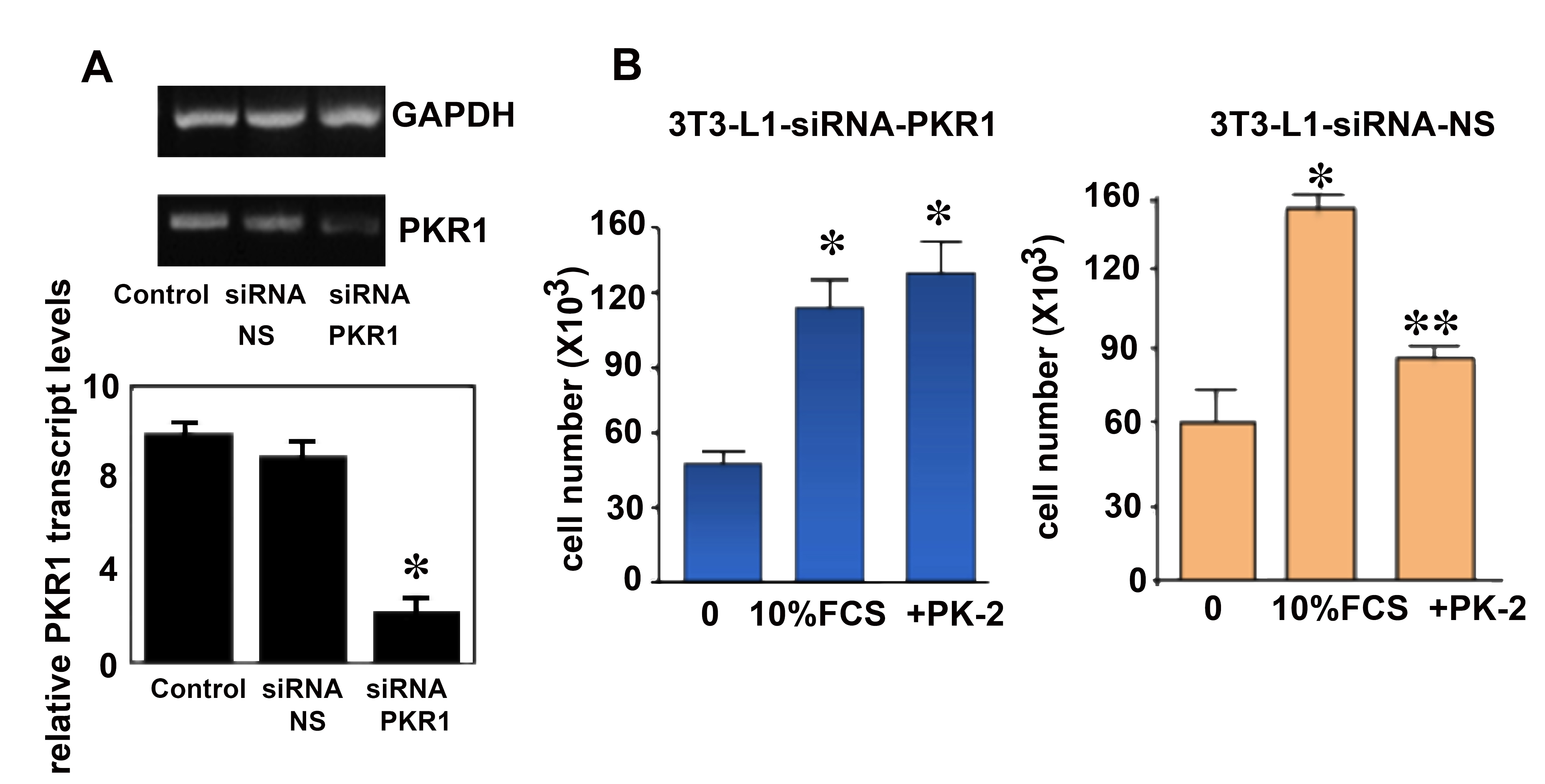

Supplement: Figure S5 — Prokineticin-2 mediated proliferation rate in 3T3-L1 cells, expressing low level of PKR1. A) Representative illustration of PCR analyses revealed that siRNA for PKR1 significantly reduced PKR1 expression 48 hours after siRNAPKR1 transfection. Histogram shows quantification of the PKR1 expression levels in each group (n = 3, *p<0.05). siRNA NS: nonspecific siRNA, siRNA PKR1: siRNA for PKR1. B) PK-2 was not able to inhibit proliferation induced by 10% FCS for 3 days in the 3T3-L1 cells transfected with siRNA for PKR1 (n = 3, *p<0.05 different then initial 0 time). In the 3T3-L1 cells transfected with siRNA-NS, PK-2 inhibits % FCS induced proliferation rate (*p<0.05 different then initial 0 time ** p<0.05 different than 10% serum). (TIF) [file pone.0081175.s005.tif]

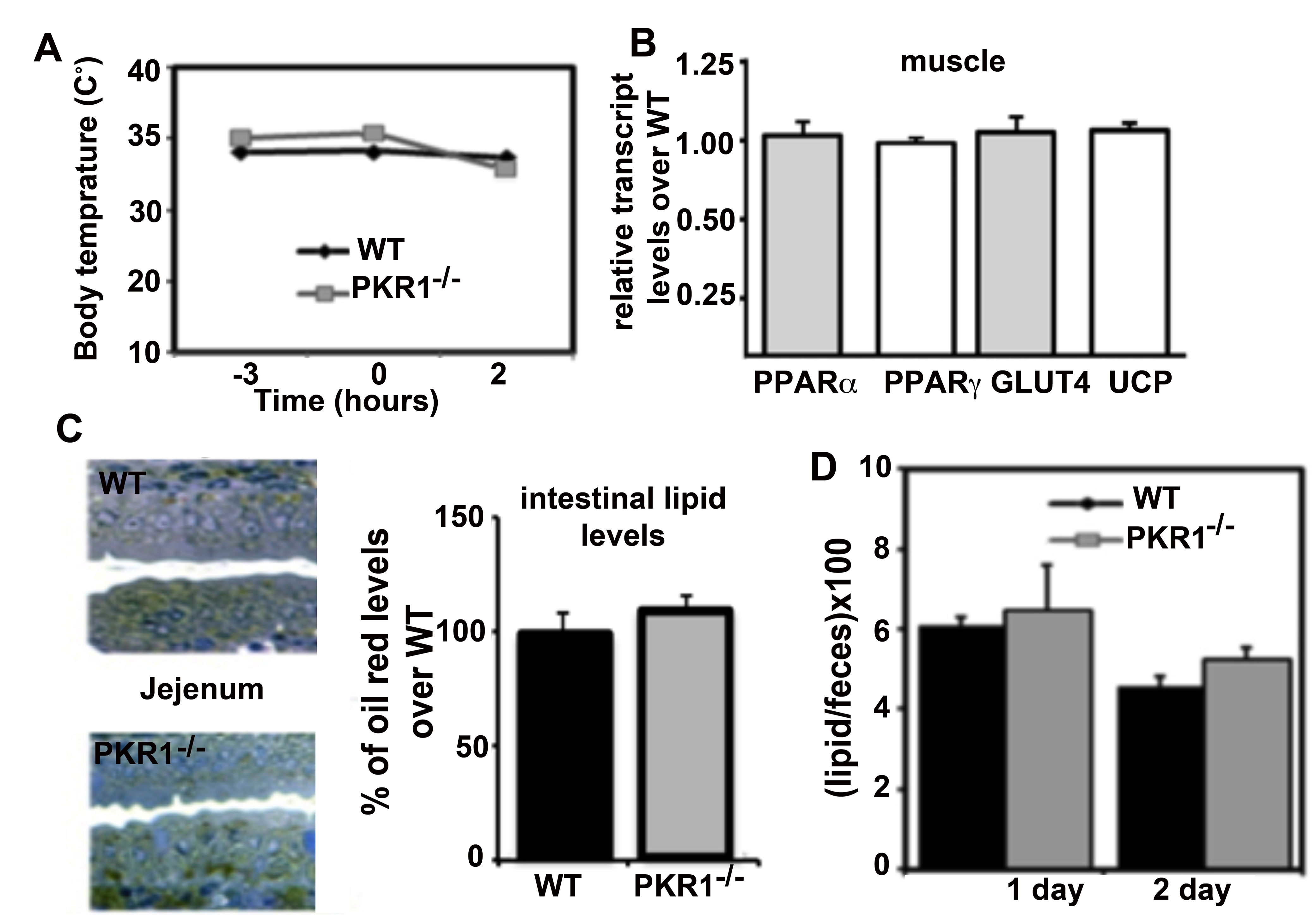

Supplement: Figure S6 — Thermogenesis, expression profiles of energy metabolism-related genes in the muscle and lipid absorption in PKR1−/− null mutant mice. A) Body temperature of PKR1-deficient (PKR1−/−) and wild (WT) mice (n = 6), 3 hours before cold exposure (room temperature), the time of cold exposure (0) and 2 hours after cold (4°C) exposure. B) Total RNA was extracted from the muscle of 40-week-old mice. qRT-PCR was performed using primers listed in Table 1. The values in the muscle were normalized with those of β-actin. No statistically significant differences were noted between WT+/+ and PKR1−/− mice (p>0.05, n = 3). PPARs, peroxisome proliferators-activated receptor; GLUT4, glucose transporter type 4; UCP, uncoupling protein. All data are presented as mean ± SEM (n = 4). C) Representative of semi-thin analyses of Jejunum derived from mutant and wild type mice 20 min after oil (vegetable oil) gavages indicating slightly higher levels of lipid absorption in the jejunum of PKR1−/− mice one hour after gavage with oil. C) Quantification of intestinal lipid levels after oil-red staining revealed a similar oil-red staining between the groups. D) The intestinal lipid/feces lipid ratio was not significantly altered between the groups (n = 6, p<0.05). (TIF) [file pone.0081175.s006.tif]

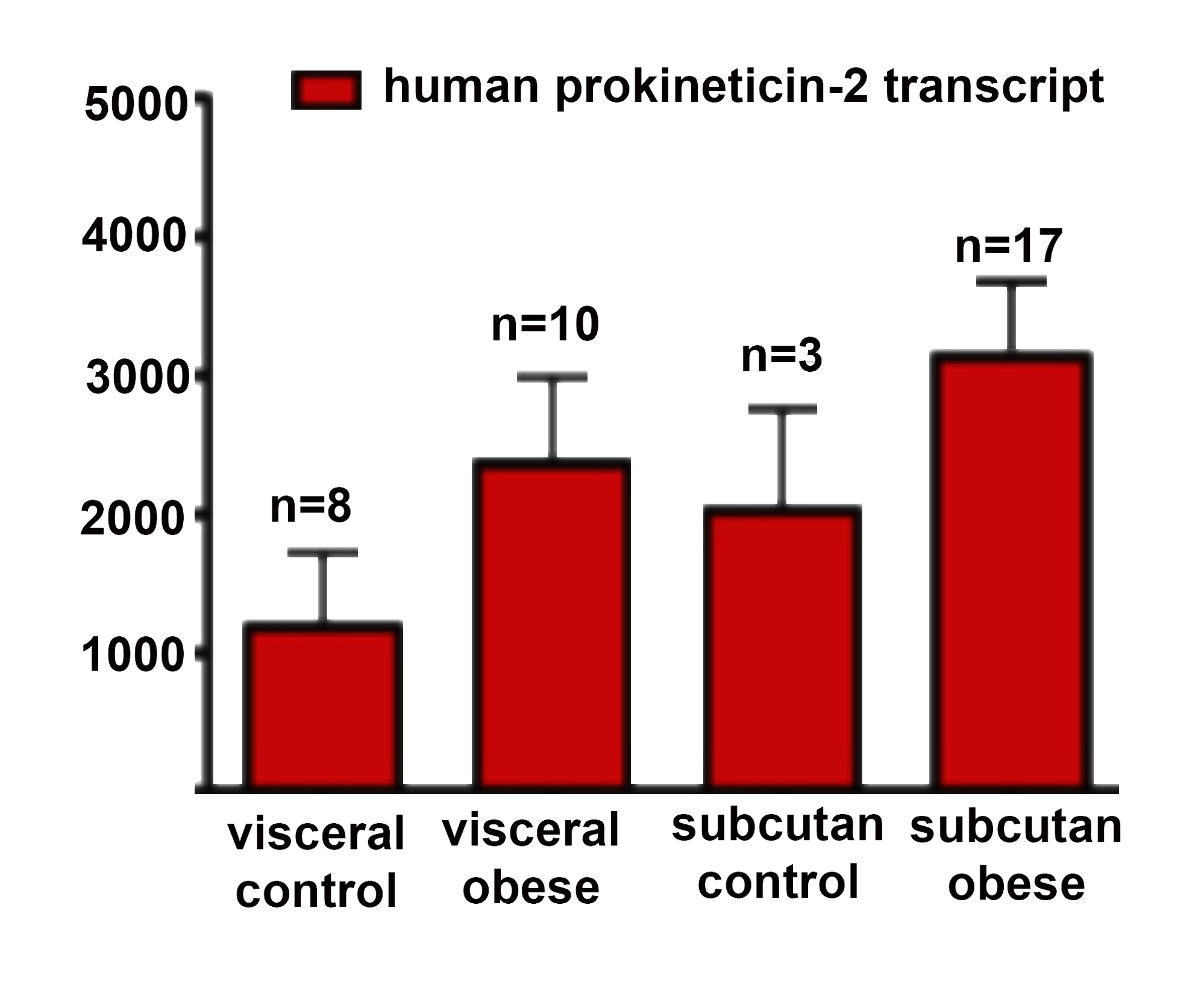

Supplement: Figure S7 — Prokineticin-2 and its receptor PKR1 expression in Human adipose tissues. Quantitative PCR analyses show transcript levels of prokineticin-2, a ligand were increased in obese human WAT tissues. (TIF) [file pone.0081175.s007.tif]
